# Strengthening rheumatology services: Rwanda's path to comprehensive training and care

**DOI:** 10.1093/rap/rkaf071

**Published:** 2025-06-05

**Authors:** Janvier Murayire, Kara L Neil, Emile Twagirumukiza, Emile Sebera, Menelas Nkeshimana, Zerihun Abebe, Marie Goretti Baransabira, Rosette Mutuyimana, Augustin Sendegeya, Xavier Chevalier, Jean Paul Rwabihama

**Affiliations:** Department of Internal Medicine, King Faisal Hospital Rwanda, Kigali, Rwanda; Department of Internal Medicine, Africa Health Sciences University, Kigali, Rwanda; Department of Internal Medicine, University of Rwanda, Kigali, Rwanda; Department of Internal Medicine, King Faisal Hospital Rwanda, Kigali, Rwanda; Department of Internal Medicine, Africa Health Sciences University, Kigali, Rwanda; Department of Internal Medicine, King Faisal Hospital Rwanda, Kigali, Rwanda; Department of Internal Medicine, Africa Health Sciences University, Kigali, Rwanda; Department of Internal Medicine, King Faisal Hospital Rwanda, Kigali, Rwanda; Department of Internal Medicine, Africa Health Sciences University, Kigali, Rwanda; Department of Health Workforce Development, Ministry of Health Rwanda, Kigali, Rwanda; Department of Internal Medicine, King Faisal Hospital Rwanda, Kigali, Rwanda; Department of Internal Medicine, Africa Health Sciences University, Kigali, Rwanda; Department of Internal Medicine, Rwanda Military Referral and Teaching Hospital, Kigali, Rwanda; Department of Internal Medicine, King Faisal Hospital Rwanda, Kigali, Rwanda; Department of Internal Medicine, King Faisal Hospital Rwanda, Kigali, Rwanda; Department of Internal Medicine, Africa Health Sciences University, Kigali, Rwanda; Department of Rheumatology, Henri Mondor University Hospital, AP-HP, Creteil, France; Department of Internal Medicine, University of Rwanda, Kigali, Rwanda; Department of Health Workforce Development, Ministry of Health Rwanda, Kigali, Rwanda

Rheumatologic conditions are among the most significant global health challenges, affecting nearly one-third of the population, particularly in the elderly [1]. These diseases are a major cause of disability in low- and middle-income countries, significantly impacting quality of life and daily functioning [1]. In sub-Saharan Africa, the gap is further exacerbated by a significant shortage of rheumatologists: fewer than 150 specialists serving nearly 1 billion people [1]. This limits access to clinical service and complicates accurate diagnosis, which often requires specialized expertise. Additionally, the lack of rheumatologists in rural areas creates additional disparities in care, making it difficult to determine the prevalence of rheumatological diseases [2]. As a result, patients may seek care from general practitioners, orthopedic specialists or even traditional healers [2].

The future of rheumatology in Africa relies on investments in training, service delivery, and care access, supported by health professional development and global academic partnerships in underserved regions. Other countries in the region, including Cameroon and Mauritius, have also highlighted the need to prioritize this training across workforce cadres [3, 4]. While training more rheumatologists is crucial, they will also require institutional support to establish services, educate both healthcare workers and the general population, and improve access to diagnostic and treatment capabilities. Targeted strategies tailored to resource-limited settings are also needed, focusing on prevention, diagnosis and cost-effective interventions. The global socioeconomic burden of rheumatologic conditions is significant. The European Fit for Work survey found that 40% of patients with musculoskeletal disorders had reduced earnings, while over 65% retired prematurely [5]. These statistics emphasize the urgent need for enhanced rheumatology services, not only in Africa but globally, to alleviate the burden on patients and healthcare systems.

Contributing to equitable access to rheumatology services, Rwanda operates a community-based health insurance scheme, which covers a significant portion of the population, ensuring broad access to primary healthcare services. However, despite this coverage, patients may still face financial barriers, particularly for specialized services such as rheumatology. Out-of-pocket costs include annual premiums, co-payments for consultations, laboratory tests, imaging studies and medication. While these costs are generally lower than in many other healthcare systems of developed countries, they can still be prohibitive for low-income patients, potentially delaying or limiting access to specialized rheumatologic care.

To address healthcare workforce gaps in Rwanda, significant efforts are underway to scale up the health workforce through the national ‘4x4’ strategy, aiming to quadruple the health workforce in four years [6]. Expected outcomes of this national strategy include increasing the ratio of healthcare workers to 4 per 1000 people by 2028, with strict observance of the quality of the training. This will lead to positive gains, including expected increased life expectancy to 80 years by 2035 [6]. With an aging population and rising life expectancy, the demand for rheumatology care is growing. Currently, no data are available on the prevalence of rheumatic diseases in Rwanda, as rheumatology is a newly introduced specialty and no direct observations have been made. Estimates are instead based on extrapolations from Sub-Saharan and East African data. In 2023, Rwanda’s first functional rheumatology unit was established at King Faisal Hospital Rwanda (KFHR) by the country’s first rheumatologist, who was trained in France. KFHR, a 160-bed tertiary teaching hospital, hosts specialized services like cardiothoracic and renal transplant surgeries, among others, and serves as a key training site for the University of Rwanda’s subspecialty fellowships, integrating clinical care with education. This article documents the process Rwanda followed in establishing the first dedicated rheumatology unit in the country, aiming to provide a framework for other limited resource settings aiming to do the same.

## Clinical service excellence

Strengthening clinical service provision was prioritized at KFHR, including setting up a dedicated outpatient unit and triage system, organizing inpatient admission procedures and building a trained interdisciplinary team (comprising nurses, rehabilitation specialists and physicians) for rheumatological diseases. A compact diagnostic ultrasonography and interventional rheumatology unit followed. To support this, the hospital laboratory was upgraded with essential immunological assays, such as rheumatoid factor, anti-cyclic citrullinated peptide, antinuclear antibodies, extractable nuclear antibodies, myositis-specific antibodies and anti-neutrophil cytoplasmic antibody, all of which are crucial for diagnosing and monitoring rheumatological conditions. Furthermore, diagnostic modalities for rare diseases are available, including human leucocyte antigen 27 and 51 testing for spondyloarthritis and Bechet’s disease, respectively, and angiotensin converting enzyme for sarcoidosis. Additionally, quality control processes for synovial fluid evaluation were implemented, ensuring diagnostic accuracy. Finally, a culture of imaging analysis by rheumatologists is encouraged to minimize potential diagnostic errors. Rheumatologists started interpreting X rays, CT scans, bone mineral density assessments and MRI studies. Beyond diagnostics, collaborations with pharmaceutical companies facilitated access to previously unavailable medications, including anti-interleukin 1 (Anakinra), anti-interleukin 6 (Tocilizumab), anti-TNF alpha (Certolizumab, Etanercept and Adalimumab), anti-CD20 (Rituximab) and Janus Kinase inhibitors (Tofacitinib). Ongoing efforts aim to make these resources affordable and accessible to all citizens.

## Partnerships and workforce development

To further address the rheumatology gap, Rwanda is scaling up clinical services and workforce development through a multi-tiered training approach. With only two national rheumatologists, the University of Rwanda launched a rheumatology fellowship in partnership with the ‘*Collège Français des Enseignants de Rhumatologie*’ (COFER) to train three fellows annually starting in 2025, ensuring the expansion of specialized care in Rwanda. This initiative is complemented by efforts to empower allied health workers, training them to perform basic procedures such as arthrocentesis and joint infiltrations, while specialized nurses manage clinics and administer treatments, thereby enhancing service quality. Globally, these professionals have been instrumental in improving patient outcomes through coordinated, cost-effective care. However, in East Africa, the shortage of rheumatologists, trained specialized allied healthcare workers and nurses has hindered comprehensive rheumatological disease management [7]. By integrating them into the care network, Rwanda aims to close this gap and strengthen service delivery [5]. Beyond clinical workforce expansion, sustaining high-quality care requires investment in educational infrastructure and faculty development. The newly established rheumatology fellowship in Rwanda plays a crucial role in this process by providing structured training that integrates clinical practice with advanced procedural skills. Additionally, ongoing professional development programs, such as short courses and international training collaborations, will ensure continuous learning and adherence to global standards of excellence in rheumatology care.

The unit’s success spurred the establishment of 11 rheumatology-oriented units in district and referral hospitals across Rwanda, managed by internal medicine physicians trained in rheumatology with intensive training with international partners further enhancing their proficiency in managing complex rheumatological cases, which are referred to KFHR as needed. Within 10 months, the expanded network treated over 1800 patients (among whom 60% are women with a mean age of 55 years), significantly surpassing the capacity of a single physician to manage such a volume. This program represents a holistic approach to rheumatology, combining infrastructure development, workforce training and international collaboration to address the rising demand for rheumatological care in Rwanda.

## Leveraging partnerships and telemedicine

Rwanda’s growing rheumatology network represents a significant step forward, but continued progress requires strengthening foundational efforts. Collaborations with international partners can enhance the diagnosis and treatment of prevalent conditions such as tendinopathies, osteoarthritis and rheumatoid arthritis while contributing to accredited, locally run training programs. These partnerships also emphasize training in advanced techniques that may not yet have sufficient local expertise, such as ultrasound-guided injections and synovial biopsies, expanding local capabilities and improving patient care. Additionally, telemedicine is emerging as a key tool in Rwanda’s rheumatology network, particularly for supporting healthcare providers in remote areas. While the country has made significant investments in improving digital health infrastructure, connectivity challenges persist, especially in rural regions. To address this, telemedicine consultations are primarily conducted from district and referral hospitals, where internet access is more reliable. This model enables patients to access specialized care without needing to travel long distances while also ensuring that local providers receive expert guidance for case management. Expanding broadband infrastructure will be crucial for optimizing the reach and effectiveness of telemedicine services in Rwanda, further strengthening the country’s efforts to enhance rheumatology care and training.

## Challenges and way forward

Rwanda faces several key challenges in strengthening rheumatology services. This primarily includes the critical shortage of rheumatologists, while at present, there are only two rheumatologists for the country’s 14 million people. With the upcoming fellowship program, 2 additional faculty will help improve the rheumatologist-to-patient ratio to approximately 1 per 5 million people. However, a major challenge remains in maintaining a steady training pipeline of 2–3 fellows per year to ensure the growth and sustainability of the workforce. Cost and accessibility of diagnostics and treatment—although testing and treatment for autoimmune and rare diseases are available, their costs remain high relative to the Rwandan GDP per capita of $1010, as reported by the World Bank [8]. Fortunately, most insurance plans cover 85% of total costs, and 92% of the population was insured as of 2025, according to the Ministry of Health Rwanda.

There is a broad range of treatments available, including conventional DMARDs (cDMARDs) and targeted DMARDs (tDMARDs). Out-of-pocket (OOP) costs vary depending on the insurance provider. Typically, insurance covers at least 85% of the total cost, resulting in monthly OOP expenses of $1–$5 for cDMARDs (except for Sulfasalazine, which is around $20) and $35–$100 for tDMARDs. As of now, predominantly subcutaneous formulations of tDMARDs are used, with rituximab as the only exception, delivered by infusion according to established protocols, since the subcutaneous variant is not yet sanctioned for rheumatoid arthritis, alongside Tofacitinib, which is an oral formulation [10]. However, if an IV biologic is required for any reason, it can be easily outsourced, typically arriving within 10 days. IV formulations are not our first-line choice and are generally reserved as a last resort. A proportion of patients have refrigerators with adjustable thermostats to maintain the required temperature for biologics. For those without proper storage, their treatment is kept at healthcare facilities, where they visit for administration. The dosing frequency ranges from every 2–4 weeks, except for rituximab (up to 20 weeks) and etanercept, which may require a repeat dose after 1 week.

Another challenge is limited awareness coupled with high consultation wait times. Since rheumatology was introduced in Rwanda in 2023, public awareness of the specialty was initially limited. However, with the expansion of rheumatology care units nationwide and increased awareness through word of mouth and social media, recognition has grown significantly. As a result, the demand for consultations has surged, resulting in a reduction of average waiting times from 8 months to 6 months. Addressing these challenges requires sustained investment in training, accessibility and infrastructure.

With only 2.7 physicians per 10 000 people across the continent, telemedicine will also be leveraged going forward to strengthen rheumatology training and care [9]. Integrating clinical-grade telepresence into the rheumatology network can enhance access to expert guidance from collaborating centers, enabling continuous knowledge exchange with these international specialists. Partnerships with global institutions will support this model, ensuring a sustainable framework for training, diagnostics and care delivery. Additionally, public awareness campaigns, expanded laboratory services, a reliable supply of novel, highly efficacious medications with fewer side effects, and training for allied health professionals are crucial to building a comprehensive rheumatology care network in Rwanda. Together, these measures promise to bridge gaps in specialist care and improve outcomes for patients with rheumatological diseases in Rwanda and the sub-Saharan region, which will contribute to the progress toward universal health coverage by 2030.

## Data Availability

No new data were generated or analysed in support of this article.
